# Sulfatide with ceramide composed of phytosphingosine (t18:0) and 2-hydroxy FAs in renal intercalated cells

**DOI:** 10.1016/j.jlr.2022.100210

**Published:** 2022-04-16

**Authors:** Keiko Nakashima, Yukie Hirahara, Taro Koike, Susumu Tanaka, Keizo Gamo, Souichi Oe, Shinichi Hayashi, Ryohei Seki-Omura, Yousuke Nakano, Chisato Ohe, Takashi Yoshida, Yosky Kataoka, Masayuki Tsuda, Tatsuyuki Yamashita, Koichi Honke, Masaaki Kitada

**Affiliations:** 1 Department of Anatomy; 2 Department of Pathology; 3 Department of Urology and Andrology, Kansai Medical University, Hirakata, Osaka, Japan; 4 Laboratory for Cellular Function Imaging, RIKEN Center for Biosystems Dynamics Research; 5 Multi-Modal Microstructure Analysis Unit, RIKEN－JEOL Collaboration Center, Kobe, Hyogo, Japan; 6 Division of Laboratory Animal Science Research Center; 7 Department of Biochemistry, Kochi University Medical School, Nangoku, Kochi, Japan

**Keywords:** kidney, sulfatide, intercalated cell, imaging MS, electron microscopic analysis, 9AA, 9-acridinylamine, CGT, ceramide galactosyltransferase, CST, cerebroside sulfotransferase, CHCA, α-cyano-4-hydroxycinnamic acid, DBA, *Dolichos biflorus* agglutinin, EC, Enzyme Commission number, FACS, fluorescence-activated cell sorting, GalCer, galactosylceramide, hFA, hydroxy FA, IMCD, inner medulla collecting duct, IMS, imaging MS, LAMP2, lysosome-associated membrane protein 2, OMCD, outer medullary collecting duct, PB, phosphate buffer, qPCR, quantitative PCR, SB1a, gangliotetraosylceramide-bis-sulfate, SM3, lactosylceramide sulfate, SM4, galactosylceramide sulphate, *Ugcg*, UDP-glucose:ceramide glucosyltransferase, V-ATPase, vacuolar H+-ATPase

## Abstract

Diverse molecular species of sulfatide with differences in FA lengths, unsaturation degrees, and hydroxylation statuses are expressed in the kidneys. However, the physiological functions of specific sulfatide species in the kidneys are unclear. Here, we evaluated the distribution of specific sulfatide species in the kidneys and their physiological functions. Electron microscopic analysis of kidneys of *Cst*-deficient mice lacking sulfatide showed vacuolar accumulation in the cytoplasm of intercalated cells in the collecting duct, whereas the proximal and distal tubules were unchanged. Immunohistochemical analysis revealed that vacuolar H^+^-ATPase-positive vesicles were accumulated in intercalated cells in sulfatide-deficient kidneys. Seventeen sulfatide species were detected in the murine kidney by iMScope MALDI-MS analysis. The distribution of the specific sulfatide species was classified into four patterns. Although most sulfatide species were highly expressed in the outer medullary layer, two unique sulfatide species of *m/z* 896.6 (predicted ceramide structure: t18:0-C22:0h) and *m/z* 924.6 (predicted ceramide structure: t18:0-C24:0h) were dispersed along the collecting duct, implying expression in intercalated cells. In addition, the intercalated cell-enriched fraction was purified by fluorescence-activated cell sorting using the anti-vacuolar H^+^-ATPase subunit 6V0A4, which predominantly contained sulfatide species (*m/z* 896.6 and 924.6). The *Degs2* and *Fa2h* genes, which are responsible for ceramide hydroxylation, were expressed in the purified intercalated cells. These results suggested that sulfatide molecular species with ceramide composed of phytosphingosine (t18:0) and 2-hydroxy FAs, which were characteristically expressed in intercalated cells, were involved in the excretion of NH_3_ and protons into the urine.

Sulfoglycolipids are a subclass of acidic glycolipids containing sulfate esters on their oligosaccharide chains. Sulfatide (also known as galactosylceramide sulphate [SM4]) is the most abundant sulfoglycolipid, in which the sulfate group is attached to the C3 position of galactose in galactosylceramide (GalCer). Sulfatide is abundant in the myelin sheath, renal tubular cells, and epithelial cells of the alimentary system (1). Sulfoglycolipids have been involved in various physiological functions through their interactions with extracellular matrix proteins, cellular adhesive receptors, the blood coagulation system, and microorganisms (2). Sulfatide is synthesized from ceramide via sequential reactions catalyzed by ceramide galactosyltransferase (CGT; Enzyme Commission [EC] number: 2.4.1.45) (3) and cerebroside sulfotransferase (CST; EC: 2.8.2.11) (4, 5). Notably, CGT-knockout mice (6, 7) and CST-knockout mice (8) showed that both GalCer and sulfatide are indispensable for myelin function.

In the kidneys, sulfatide is highly expressed in the distal nephron segments and medulla (9). In addition to sulfatide, more complex sulfoglycolipids, such as lactosylceramide sulfate (SM3) and gangliotetraosylceramide-bis-sulfate (SB1a), have been identified in the kidneys (1). In contrast to sulfatide, SM3 is mainly present in the cortex and, to a lesser extent, in the medulla, whereas SB1a is distributed throughout all parts of the kidneys (10). Tissue-specific sulfoglycolipid structures are thought to result from the selective expression of specific glycosyltransferases that synthesize the neutral oligosaccharide backbone. The kidneys of *Cgt*-deficient mice lack sulfatide because of loss of the precursor GalCer; however, the levels of SM3 and SB1a, whose biosynthetic pathways circumvent the ceramide galactosylation reaction, are increased by 2–3-fold, which may partially compensate for sulfatide (11). By contrast, *Cst*-deficient mice do not express any sulfoglycolipids in the kidneys (12), and the mutant kidneys were initially thought to appear normal (8). However, Stettner *et al.* (13) subsequently performed intensive analyses on mice deficient in *Cst* and UDP-glucose:ceramide glucosyltransferase (*Ugcg*) in paired box gene 8 (Pax8)-expressing renal cells; they observed a lower urinary pH accompanied by lower ammonium (NH_4_^+^) excretion in *Cst*-deficient kidneys. Using acid load experiments, they found that sulfatides may play important roles in renal ammonium processing, urine acidification, and acid-base homeostasis (13). However, the molecular mechanisms underlying the pathological phenotype have not yet been elucidated.

The kidneys play central roles in the regulation of blood pH maintenance. In response to metabolic acidosis, excess protons in the blood are buffered by bicarbonate synthesized during the renal glutaminolysis process, and ammonium ions are excreted into the urine (14). This physiologically relevant ammoniagenesis process, which occurs in the proximal tubule (14), is preserved in *Cst*-deficient kidneys (13). The majority of luminal ammonium ions secreted from the proximal tubule are reabsorbed at the thick ascending limb of Henle’s loop and secreted again from the collecting duct, a process that involves parallel proton and NH_3_ secretion (15). The epithelium of the collecting duct is composed mainly of two different types of cells: principal cells and intercalated cells (16, 17, 18, 19). The Rhesus glycoproteins, Rhbg and Rhcg, which act as ammonia transporters, are expressed on the plasma membranes of intercalated cells (15). NH_3_ in the interstitium is transported across the basolateral membrane through both Rhbg and Rhcg. Most basolateral NH_4_^+^ uptake is mediated by Na^+^-K^+^-ATPase, where NH_4_^+^ is substituted for K^+^. Incorporated NH_4_^+^ is dissociated into NH_3_, and protons and intracellular NH_3_ are secreted into the lumen across the apical membrane through Rhcg. Protons secreted by H^+^-ATPase and H^+^-K^+^-ATPase combine with luminal NH_3_ to form NH_4_^+^, neutralizing urinary pH (15). Stettner *et al.* (13) stated that “transepithelial NH_3_ and NH_4_^+^ inward as well as proton outward movements showed similar rates in intracellular alkalinization and pH recovery, suggesting unaltered luminal NH_3_ entry and proton secretion by *Ugcg/Cst*-deficient outer medullary collecting duct (OMCD) intercalated cells,” interpreting the result of the in vitro microperfusion experiment using OMCD cells. However, the initial alkalinization phase reflecting NH_3_ entry and formation of intracellular NH_4_^+^ is remarkably suppressed in *Ugcg/Cst*-deficient OMCD intercalated cells and the subsequent acidification phase reflecting NH_4_^+^ entry, and dissociation of NH_3_ and H^+^ is preserved in *Ugcg/Cst*-deficient cells, as seen in the experiment of *Rhcg*-deficient mice (20), which was demonstrated using the same experimental procedure. Proton secretion is expected to follow the latter phase in which H^+^ is formed. Therefore, we predicted that NH_3_ excretion through Rhcg is deteriorated in sulfatide-lacking mice.

Glycosphingolipids comprise various molecular species with different lipid and carbohydrate moieties. Both the long-chain base (also referred to as the sphingoid base) and the N-acyl chain of ceramide can vary with regard to alkyl chain length, hydroxylation, and desaturation. Characterization of the ceramide moieties of sulfatide from rat renal tubule cells by MALDI time-of-flight MS analysis revealed that sulfatide contains ceramides possessing sphingosine (4-sphingenine, d18:1) with C20:0 hydroxy FA (hFA; C20:0h), C21:0h, C22:0h, C23:0, C23:0h, C24:0, C24:0h, and C24:1h as well as phytosphingosine (4-hydroxysphinganine, t18:0) with C22:0h and C24:0h (21). Marsching *et al.* (22) quantitatively measured sulfatide species in the mouse kidney using MALDI-based imaging MS (IMS) and detected sulfatide species that contain ceramide possessing sphingosine (d18:1) with FAs C16:0, C18:0, C20:0, C20:0h, C22:0, C22:0h, C24:0, C24:0h, and C26:0. Moreover, a list of 52 sulfatide species in human renal cell carcinoma was identified by MALDI Orbitrap MS (23). Thus, various molecular species of sulfatide are present in the kidney, although the physiological functions of specific sulfatide species are still unclear.

Recent advancements in IMS have revealed the characteristic distributions of specific molecular species of lipids among different tissues or cells (24). In fact, we have analyzed sulfated glycolipids in the glial developmental process (25), pharmacokinetics in an Alzheimer's mouse model (26), and phospholipid localization in the retinal layer in an optic nerve injury model (27) using iMScope MALDI-IMS analysis with high spatial resolution. Accordingly, in this study, we used an IMS approach to evaluate the morphological abnormalities in the kidneys of *Cst*-deficient mice and assess the involvement of specific molecular species of sulfatide in biological processes in the kidneys.

## Materials and methods

### Antibodies and other reagents

Fluorescein *Dolichos biflorus* agglutinin (DBA; catalog no.: FL-1031) was purchased from Vector Laboratories (CA). Anti-ATP6V0A4 rabbit IgG (catalog no.: ab204737) and anti-lysosome-associated membrane protein 2 (LAMP2) rat IgG (catalog no.: ab13524) were purchased from Abcam (Cambridge, UK). Carboxymethyl cellulose sodium salt and isoflurane were purchased from Wako (Osaka, Japan). Alexa Fluor 488- and 594-conjugated anti-rabbit IgG were purchased from Thermo Fisher Scientific (Waltham, MA). Anti-calbindin D-28K rabbit IgG (catalog no.: AB1778; Research Resource Identifier: AB_2068336) was purchased from Merck Millipore (Burlington, MA). 9-Acridinylamine (9AA), galactocerebrosides from bovine brain (catalog no.: C4905), and α-cyano-4-hydroxycinnamic acid (CHCA; catalog no.: C2020) were purchased from Merck KGaA (Darmstadt, Germany). Hoechst 33258 was purchased from Nacalai Tesque (Kyoto, Japan). RNeasy Mini Kit and Rotor-Gene platform were purchased from Qiagen (Venlo, The Netherlands). ReverTra Ace quantitative PCR (qPCR) RT master mix with genomic DNA remover and Thunderbird qPCR Mix were purchased from TOYOBO (Osaka, Japan).

### Animal care

All animal experiments were conducted in strict accordance with the institutional guidelines of the National Institutes of Health and the Guide for the Care and Use of Laboratory Animals of the National Institutes of Health Animal Care. Maintenance and surgeries were performed in full compliance with the regulatory standards for the Animal Research Facilities of the Animal Ethics Committee of Kansai Medical University (Hirakata, Japan; approval ID: 20-086). C57BL6J and TgH (CST-neo) mice (8) were housed in plastic cages with standard bedding and continuous access to food and water. The temperature was maintained at 22°C under standard light conditions with a 12 h light/dark cycle.

### Tissue preparation

Kidneys of 10-week-old female C57BL6 and TgH (CST-neo) mice (8) were quickly dissected. Unfixed tissues were embedded in cold 2% carboxymethylcellulose on dry ice and sliced to a thickness of 10 μm using a cryostat (CM3050 S; Leica, Nussloch, Germany). The sections were mounted on glass slides coated with indium tin oxide (SI0100N) for IMS and microslide glass (CRE-02; Matsunami Glass, Kishiwada, Japan) for immunofluorescent labeling. Hoechst 33258 was purchased from Nacalai Tesque.

### Immunofluorescent labeling and confocal microscopy

Serial sections of tissues for IMS were fixed with 4% formaldehyde, washed in 0.1 M phosphate buffer (PB) and immunostained with anti-ATP6V0A4 (1:200 dilution), anticalbindin (1:200 dilution) antibodies, anti-LAMP2 (1:200), fluorescein DBA (1:100 dilution) diluted in 0.1 M PBS containing 0.1% Triton X-100 for 12 h. After rinsing with PBS, the sections were incubated with Alexa Fluor 594- or Alexa Fluor 488-conjugated secondary antibodies (1:1,000 dilution) for 1 h, washed with 0.1 M PBS, and mounted with a medium containing 100 mM dithiothreitol, 5 μg/ml Hoechst 33258, 50% glycerol, and PBS (pH 7.4). Images were obtained using a confocal laser-scanning microscope (LSM700; Carl Zeiss, Jena, Germany). Pearson’s correlation coefficient (*r*) for the quantitative analysis of colocalization was obtained by performing pixel intensity correlations over space using ImageJ (Fiji)'s Coloc 2 system (National Institutes of Health, Bethesda, MD).

### IMS analysis with MALDI by iMScope

Mouse kidneys were embedded in 2% carboxymethylcellulose on dry ice and sliced to 10 μm thickness using a cryostat. The sections were mounted on glass slides coated with indium tin oxide (Matsunami Glass). Sliced samples were vapor-deposited using 9AA as a negative mode matrix and CHCA as a positive mode matrix under dry vacuum conditions. Sublimation of 9AA and CHCA was performed at 220°C for 9 min and 250°C for 15 min, respectively, using SVC-700TMSG/7PS80 vacuum vapor deposition equipment (Sanyu Electron, Tokyo, Japan).

IMS was performed using an iMScope MALDI-mass spectrometer (Shimadzu, Kyoto, Japan) in the negative and positive reflection mode. To acquire mass spectra, the samples were scanned using a 1,000 Hz Nd:YAG laser with 50 accumulating shots. The detector voltage and sample voltage were 1.66 and 3 kV, respectively. We collected spectra over the *m/z* range from 600 to 1,000 at a scan pitch of 10–25 μm, and the intensity of the iMScope laser was set to 46. The data were analyzed using the IMS Solution software package (Shimadzu). After IMS analysis, the section was washed by PBS to remove the deposited matrix and stained with hematoxylin and eosin according to the general method.

### Electron microscopy

Mice were deeply anesthetized by intraperitoneal injection of a mixture of anesthetics (0.15 mg/kg medetomidine hydrochloride, 2 mg/kg midazolam, and 2.5 mg/kg butorphanol tartrate) and perfused with 0.1 M PB followed by 2% formaldehyde and 2% glutaraldehyde in 0.1 M PB. Kidneys were sectioned at 2 mm thickness, immersed in the same fixative for 6 h at 4°C, and postfixed with 2% osmium tetroxide in 0.1 M PB at 4°C. Tissues were then embedded in epoxy resin, and sections of 80 nm thickness were prepared. The sections were mounted on silicon wafers, stained with 1% uranyl acetate for 15 min, and then stained with Sato’s lead staining solution for 5 min (28). The cells were imaged using scanning electron microscopy (JSM-7800F; JEOL Ltd, Tokyo, Japan). Backscattered electrons were detected, and black and white were reversed.

### Fluorescence-activated cell sorting

Eight-week-old C57BL/6 mice were perfused with PBS for 5 min, and blood was removed completely. The kidneys were dissected out, and the capsule was removed and cut into small pieces in HBSS. The tissues were then treated with 0.5% collagenase and DNase I by shaking at 37°C for 2 h. The suspension was centrifuged for 5 min at 1,500 rpm at 4°C. After removal of the supernatant, the remaining tissue was washed twice in HBSS and suspended in 2 ml HBSS. The tissues were then dissociated by trituration. The resulting cell suspension was filtered through a 70 μm nylon cell strainer. To isolate the intercalated cells, fluorescence-activated cell sorting (FACS) was performed using a FACS Aria instrument (BD Biosciences, Franklin Lakes, NJ) with antibodies targeting ATP6V0A4, a marker of intercalated cells. After treatment with mouse kidney-derived collagen, the cells were collected and labeled with anti-ATP6V0A4 antibodies (1:200 dilution) on ice for 15 min. The cells were then collected by centrifugation for 5 min at 15,000 rpm and 4°C, washed with PBS, and colabeled with Alexa Fluor 488-labeled anti-rabbit IgG on ice for 15 min. The cells were sorted by forward scatter and side scatter. After centrifugation for 5 min at 1,500 rpm at 4°C, methanol was added, and the cells were mechanically disrupted using a BioMasher (Nippi Incorporated, Tokyo, Japan). The lysed solution was dropped onto a metal plate and analyzed using IMS.

### RT-qPCR

Total RNA was extracted from FACS-purified vacuolar H^+^-ATPase (V-ATPase) 6V0A4-positive cells using an RNeasy Mini Kit. Complementary DNA was reverse-transcribed using a ReverTra Ace qPCR RT master mix with genomic DNA remover. qPCR was then performed using a Rotor-Gene platform and Thunderbird qPCR Mix. Primer sequences corresponding to each gene used for PCR are listed in Table 1. A thermal cycler, with the following settings: one cycle at 95°C for 1 min, followed by 40 cycles of denaturation at 95°C for 10 s and extension at 60°C for 60 s. The 2^−ΔΔCt^ method was used to calculate relative target gene expression levels (29) using hypoxanthine phosphoribosyltransferase 1 as a housekeeping gene. PCR with all complementary DNA samples was performed in duplicates.

**Table 1:** Primer sequences

	Primer name	Forward primer/reverse primer
1	Degs2_300F	5′-GCATCAACCACTCGCTGACA-3′
	Degs2_381R	5′-CGGTTTCGGGAGACACAACT-3′
2	Atp6v0a4_2253F	5′-AGCCAAGCACCAGAAATCTCA-3′
	Atp6v0a4_2321R	5′-GAGTGGTCACCCTCCACAGC-3′
3	Slc26a4_ 2706F	5′-AGGCACCACACAAGAAACAC-3′
	Slc26a4_ 2829R	5′-TGCAGCACATCCGAACATTG-3′
4	Slc4a1_1093F	5′-AGGACCTGGTGTTGCCAGAG-3′
	Slc4a1_1243R	5′-CGGTTATGCGCCATGGA-3′
5	Fa2h_660F	5′-GCTCTTCGCATCACTCACAAG-3′
	Fa2h_742R	5′-AAAGCATGCCCAGCACAAAG-3′
6	Hprt_1009F	5′-CTCTGGTAGATTGTCGCTTATCTTGTAAG-3′
	Hprt_1237R	5′-CCTCTTAGATGCTGTTACTGATAGGAAATC-3′
7	B2m_F	5′-TGGTGCTTGTCTCACTGACC-3′
	B2m_R	5′-CCGTTCTTCAGCATTTGGAT-3′
8	Gapdh_F	5′-AACTTTGGCATTGTGGAAGG-3′
	Gapdh_R	5′-ACACATTGGGGGTAGGAACA-3′

### Human samples

The study of human samples was approved by the Institutional Review Board of Kansai Medical University, and informed consent was obtained from each patient (protocol number: 2020308). All study protocols were consistent with the recommendations of the Declaration of Helsinki as a statement of ethical principles for medical research involving human subjects.

### Statistical analysis

The average signal intensity of the region of interest (250 × 250 μm) in three different areas of the outer medulla from each animal was analyzed using ImageJ software. Comparison of the signal intensity of immunostaining in wild -type and *CST*-null mice was performed by Student’s *t-*test. Values represent mean standard deviations of signal intensity from three adult animals per group.

## Results

### *Cst*-deficient mice showed abnormalities in intercalated cells of the collecting duct

**Fig. 1:**
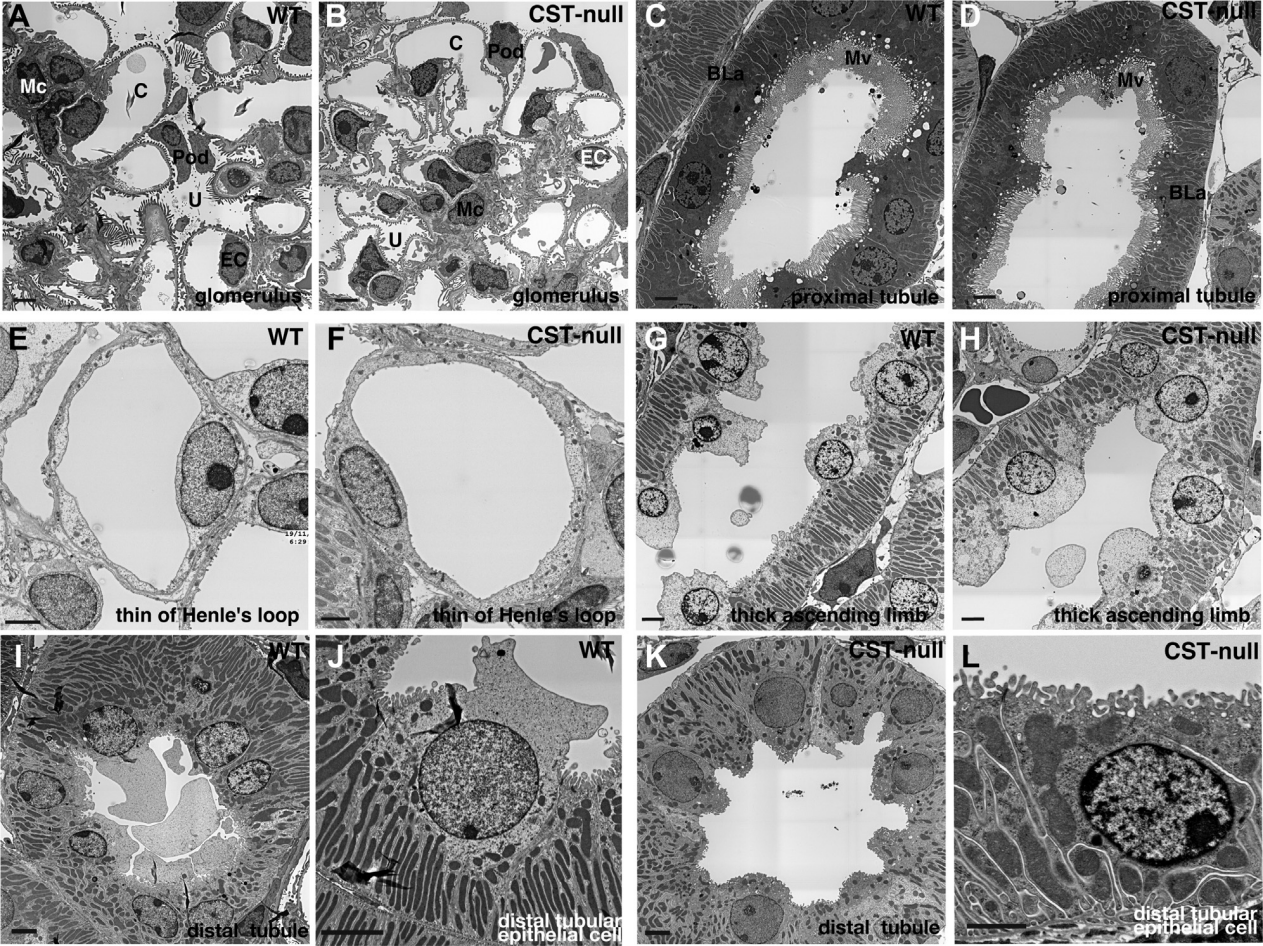
Electron microscopic analysis of the glomerulus, proximal tubules, Henle’s loop, and distal tubules of *Cst*-null mice. There were no morphological changes in the glomerulus (A, B), proximal tubules (C, D), Henle’s loop (E, F), and ascending limb (G, H) between wild-type (A, C, E, and G) and *Cst*-null mice (B, D, F, and H). The distal tubular epithelial cells (I–L) showed no morphological changes between wild-type (I, J) and *Cst*-null mice (K, L). N = 3. The scale bar represents 2 μm. Representative data are shown. BLa, basal lamina; C, capillary lumen; EC, endothelial cell; Mc, mesangial cell; Mv, microvillus; Pod, podocyte; U, urinary space.

**Fig. 2:**
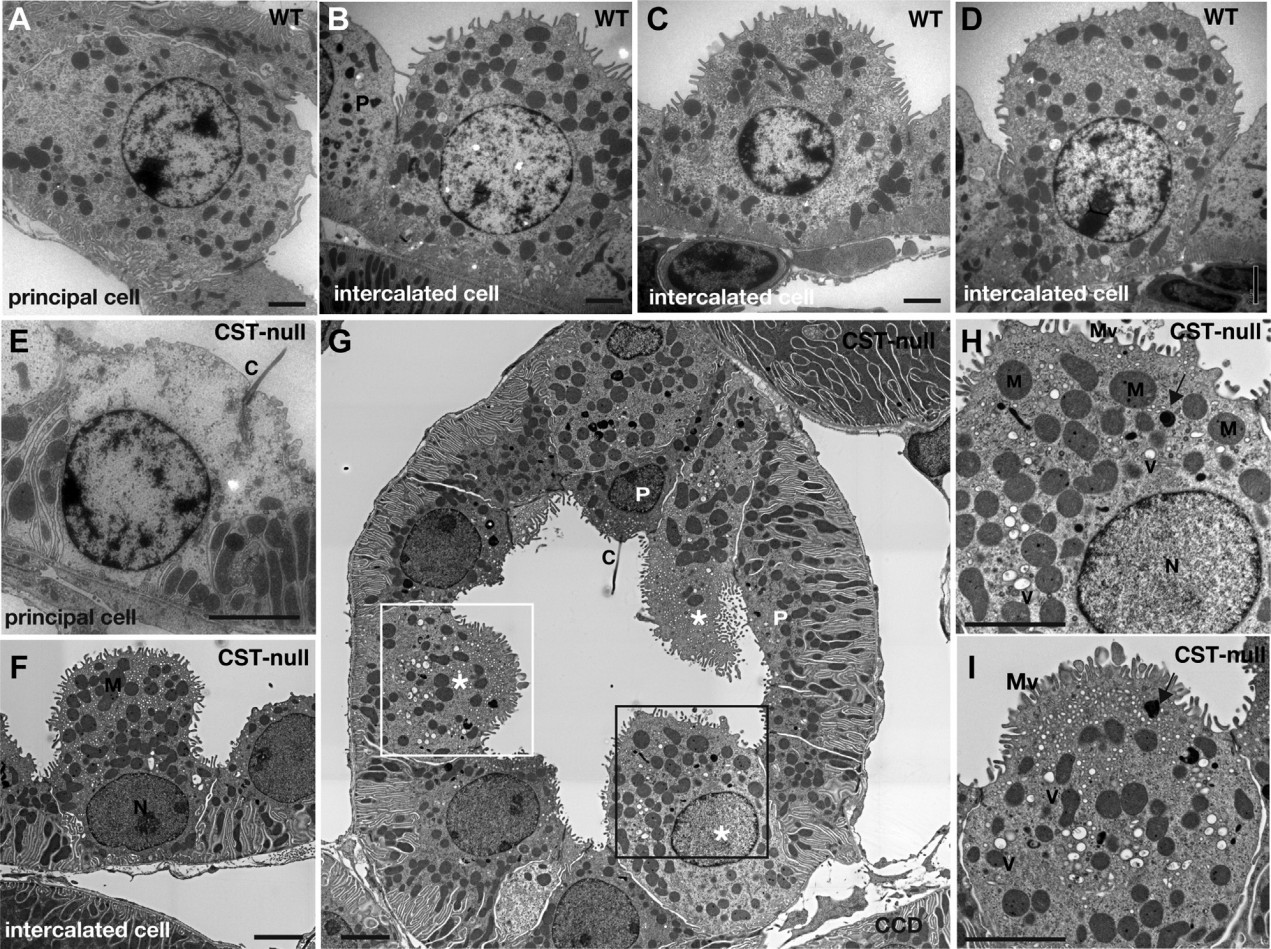
Electron microscopic analysis of the collecting tubules of *Cst*-null mice. The intercalated cells of the CCD in *Cst*-null mice (F–I) compared with wild-type mice (B–D). H and I are magnified images of the field of view of the black and white squares in G, respectively. There were many vesicle and lipid droplets (arrows in H and I) in the cytoplasm compared with that in wild-type mice (B–D). Principal cells in *Cst*-null mice (E and P in G) did not show any abnormalities compared with those in wild-type mice (A). Stars and P in G show intercalated cells and principal cells, respectively. Three representative intercalated cells are shown. N = 3. The scale bar represents 2 μm. Arrows, lipid droplets; C, cilia; M, mitochondria; Mv, microvillus; N, nucleus; V, vesicle.

**Fig. 3:**
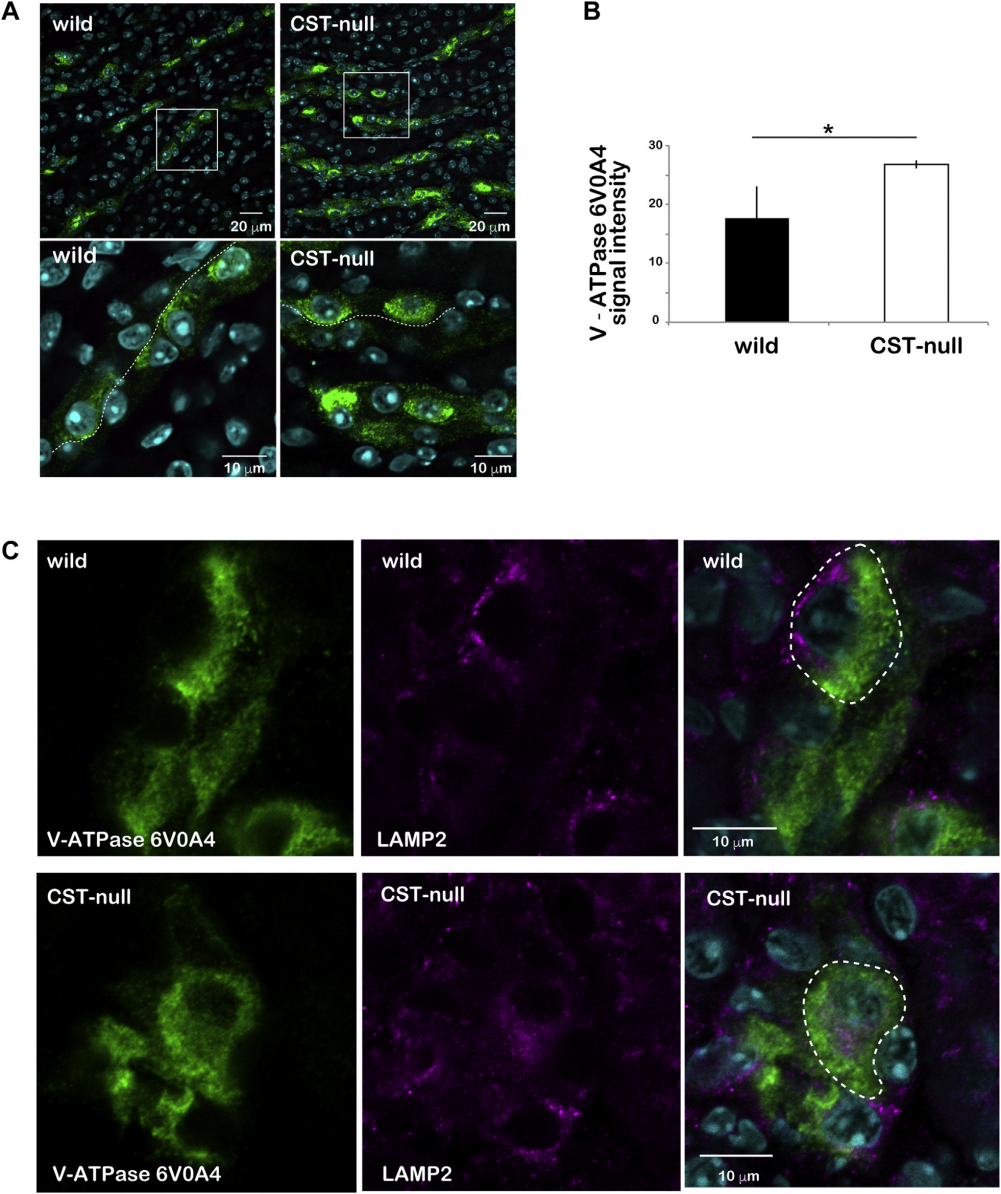
Immunohistochemical analysis of the collecting tubules of *Cst*-null mice. Comparison of the expression of V-ATPase 6V0A4 in intercalated cells of wild-type and *Cst*-null mice (A, B). Immunohistochemical imaging of V-ATPase 6V0A4 (green), a marker of intercalated cells, in the collecting ducts (A). V-ATPase 6V0A4 immunostaining images from wild-type and *Cst*-null mice are shown. Magnified images of view in the white squares in the upper panels are shown in the lower panels (A). The nucleus (blue) is stained with Hoechst 33258. Thin dotted lines show the tubule lumen (A). Comparison of the signal intensity of V-ATPase 6V0A4 immunostaining in wild-type and *Cst*-null mice (B). Significant differences were determined using Student’s *t*-tests. Values represent means ± standard deviations of the ratio of the signal intensity from three adult male mice. ∗*P* < 0.05. The average signal intensity of the region of interest (250 × 250 μm) in three different areas of the outer medulla from each animal was analyzed using ImageJ software. Double immunostaining for the lysosomal marker LAMP2 and V-ATPase 6V0A4 from wild-type and *Cst*-null mice (C). The Pearson's coefficients for the quantitative analysis of colocalization in the area are enclosed by a white dotted line.

To examine morphological changes caused by the loss of sulfatide, we compared kidney tissues from wild-type and *Cst*-null mice by electron microscopy. No morphological abnormalities were observed in the slit structure of podocytes that made up the glomerulus (Fig. 1A, B), structures of microvilli and basal lamina in the proximal tubules (Fig. 1C, D), Henle’s loop (Fig. 1E, F), basal infolding in the thick ascending limb (Fig. 1G, H), and distal tubule (Fig. 1I–L). The cortex collecting duct was composed of principal cells with cilia characterized by low infolding near the base of the cell (Fig. 2A, E, and P in G) and intercalated cells protruding toward the lumen without cellular interdigitation and basal infolding (Fig. 2B–D, F-I). It has been known to appear from the connective tubules to the outer medullary collecting ducts (16, 17, 18, 19). Electron microscopy analysis of the cortex collecting duct of *Cst*-null mice (Fig. 2E–I) revealed abnormalities in the intercalated cells protruded into the lumen, which accumulated many vesicles and lipid droplets in the cytoplasm compared with that of wild-type mice (Fig. 2B–D). Because accumulation of intracellular vesicles was found in the intercalated cells of *Cst*-null mice, the distribution of the intercalated cell-specific V-ATPase, which is localized in the apical plasma membrane in intercalated cells (16, 17), was investigated using immunohistochemistry. V-ATPase 6V0A4 subunit-positive granules were predominantly localized in the cytoplasm of *Cst*-null intercalated cells, whereas these were confined to the apical side in wild-type intercalated cells (Fig. 3A). The intracellular signal intensity of V-ATPase 6V0A4 was significantly higher in *Cst*-null mice than in wild-type mice (Fig. 3B). These sections were stained for a lysosomal marker LAMP2 to ascertain whether the accumulated V-ATPase-containing vesicles represent lysosomes. A part of intracellular V-ATPase was overlapped with LAMP2 in *Cst*-null mice, while no overlapping was observed in wild-type mice (Fig. 3C). The colocalization between V-ATPase and LAMP2 was analyzed using the Pearson’s correlation coefficient. The Pearson’s correlation coefficient (*r*) was 0.32 for *Cst*-null mice and 0.02 for wild-type mice. Therefore, a portion of V-ATPase colocalizes with lysosomes in *Cst*-null mice, suggesting that the increased V-ATPase-positive vesicles in *Cst*-null mice are lysosomes.

### Seventeen sulfatide molecular species were detected in mouse kidneys

**Fig. 4:**
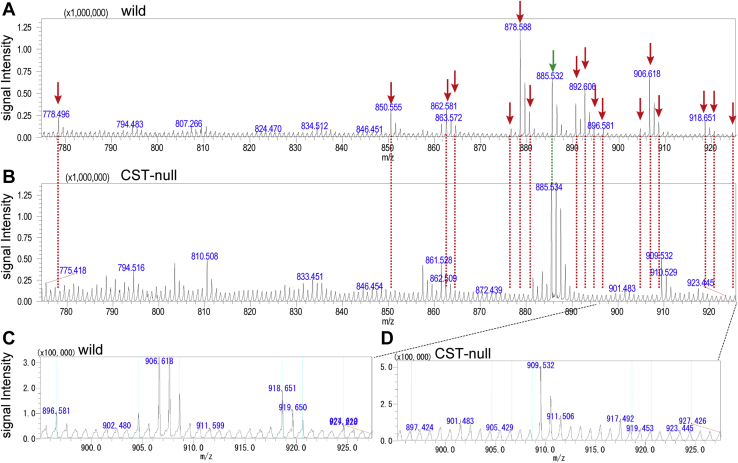
Comparison of the mass spectra of the major sulfatide molecular species in the kidneys of wild-type and *Cst*-null mice. Averaged negative ion MALDI spectra of mouse kidneys (A, B). The red arrows indicate the 17 major sulfatide species, and the green arrows indicate phosphatidylinositol *m/z* 885.53 as an internal standard. In order from left to right: *m/z* 778.51, 850.57, 862.60, 864.58, 876.58, 878.60, 880.61, 890.63, 892.61, 894.63, 896.61, 904.61, 906.63, 908.65, 918.67, 920.63, and 924.64 (A, B). Spectra for kidneys from *Cst*-null mice (B, D). The spectra of all 17 major sulfatides completely disappeared, whereas the phosphatidylinositol signal remained unchanged (B). Magnification of the spectra in (A, B) at the *m/z* 895.0–927.5 range (C, D). The light blue line shows the seven sulfatide species. In order from left to right: *m/z* 896.61, 904.61, 906.63, 908.65, 918.67, 920.63, and 924.64 (C, D).

To investigate the specific sulfatide molecular species present in mouse kidneys, iMScope MALDI-IMS analysis was performed on wild-type and *Cst*-null mice. Based on a previous report on sulfoglycosphingolipids in renal cells (23), analyses were performed in negative mode with 9AA as a matrix. The sulfatide composition of the whole mouse kidney was compared with that described in previous studies (21, 22, 23) and the Human Metabolome Database (http://www.hmdb.ca/). The observed mass value was compared with the mass value <0.04 differences to reference *m/z*. Wild-type kidneys showed 17 major signals that seemed to be derived from sulfatide species (*m/z* 778.51, 850.57, 862.60, 864.58, 876.58, 878.60, 880.61, 890.63, 892.61, 894.63, 896.61, 904.61, 906.63, 908.65, 918.67, 920.63, and 924.64; Table 2 and Fig. 4). Because of the absence of their spectra in the *Cst*-null kidney, all 17 signals were assigned as sulfatide species (Fig. 4). We then further characterized these 17 sulfatide species in our subsequent analyses.

**Table 2:** The exact mass values of the main sulfatide molecular species in the kidneys

Ceramide structure of sulfatide	Elemental composition	*m/z* of Precursor ［M − H］^-^
d18:1-C16:0	C40H77NO11S	778.51
d18:1-C20:0h	C44H85NO12S	850.57
d18:1-C22:0	C46H89NO11S	862.60
d18:1-C21:0h	C45H87NO12S	864.58
d18:1-C22:1h	C46H87NO12S	876.58
d18:1-C22:0h	C46H89NO12S	878.60
d18:0-C22:0h	C46H91NO12S	880.61
d18:1-C24:0/d20:1-C22:0	C48H93NO11S	890.63
d18:1-C23:0h	C47H91NO12S	892.61
d18:0-C23:0h	C47H93NO12S	894.63
t18:0-C22:0h	C46H91NO13S	896.61
d18:1-C24:1h	C48H91NO12S	904.61
d18:1-C24:0h	C48H93NO12S	906.63
d18:0-C24:0h	C48H95NO12S	908.65
d20:1-C24:0	C50H97NO11S	918.67
d20:0-C24:0	C50H99NO11S	920.63
t18:0-C24:0h	C48H95NO13S	924.64

### Distribution of specific sulfatide species classified into four patterns

**Fig. 5:**
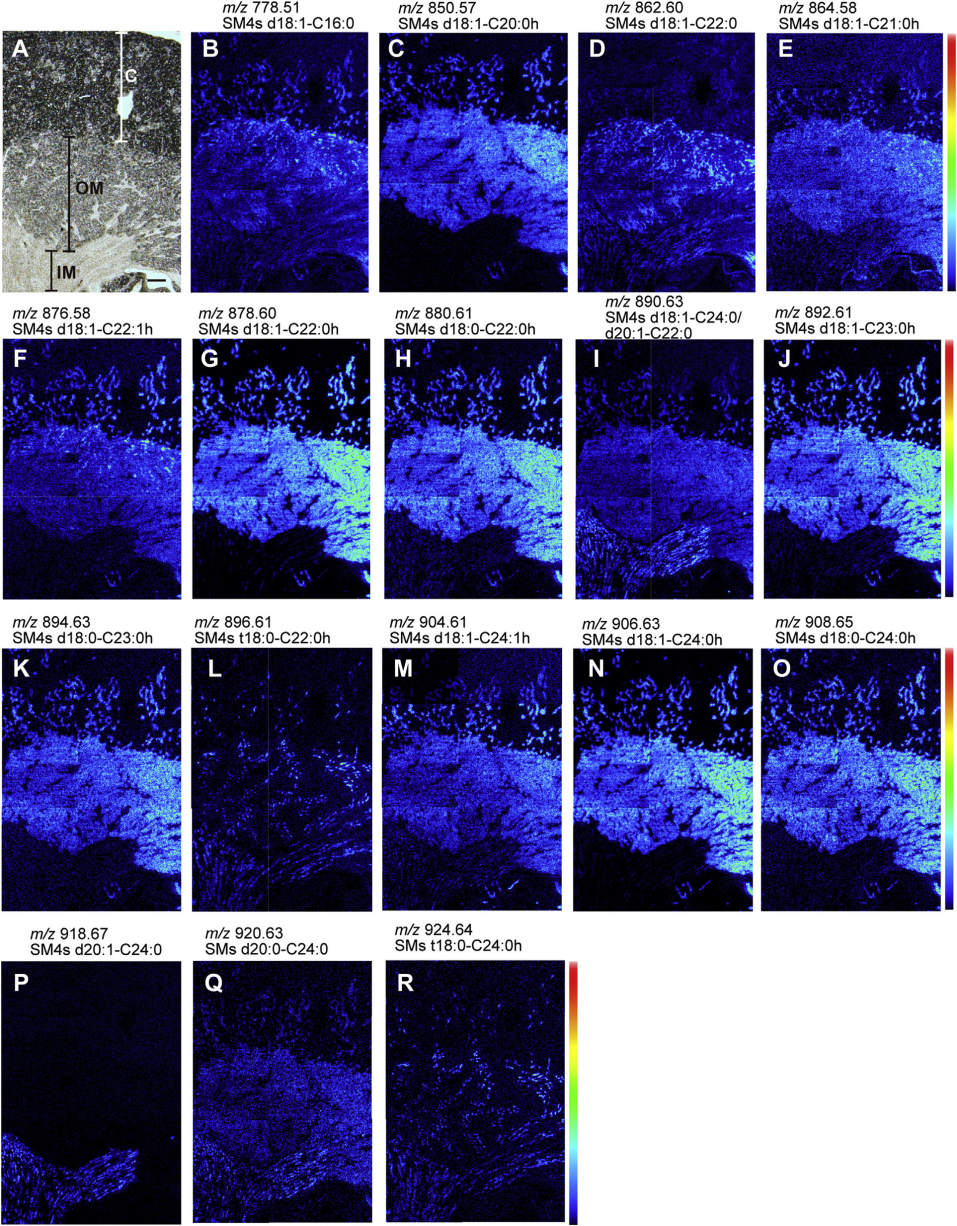
Image of sulfatide species in the kidneys of mice from iMScope data. Four types of patterns were observed. Pattern I: expressed in a part of the cortex and the entire medulla, including *m/z* 778.51 (B), 862.60 (D), 890.63 (I), and 920.63 (Q). Pattern II: expressed in a part of the cortex and the outer medulla, including *m/z* 850.57 (C), 864.58 (E), 876.58 (F), 878.60 (G), 880.61 (H), 892.61 (J), 894.63 (K), 904.61 (M), 906.63 (N), and 908.65 (O). Pattern III: limited expression in the cortical and medullary collecting ducts, including *m/z* 896.61 (L) and 924.64 (R). Pattern IV: expressed only in the inner medulla, including *m/z* 918.67 (P). An optical image of normal mouse kidney tissue (A). The color scale indicates the intensity of the signal from black (low or no signal) to red (strongest signal). The scale bar represents 200 μm. C, cortex; IM, inner medulla; OM, outer medulla.

Histology-directed IMS of the mouse kidney demonstrated that sulfatide species were present in the cortex, outer medulla, and inner medullary regions (Fig. 5). The distributions of specific sulfatide species were classified into four patterns (Table 3), as follows: pattern I, expressed in a part of the cortex and the entire medulla, including *m/z* 778.51 (predicted ceramide structure: d18:1-C16:0), 862.60 (d18:1-C22:0), 890.63 (d18:1-C24:0 and d20:1-C22:0), and 920.63 (d20:0-C24:0; Fig. 5B, D, I, Q); pattern II, expressed in a part of the cortex and the outer medulla, including *m/z* 850.57 (d18:1-C20:0h), 864.58 (d18:1-C21:0h), 876.58 (d18:1-C22:1h), 878.60 (d18:1-C22:0h), 880.61 (d18:0-C22:0h), 892.61 (d18:1-C23:0h), 894.63 (d18:0-C23:0h), 904.61 (d18:1-C24:1h), 906.63 (d18:1-C24:0h), and 908.65 (d18:0-C24:0h; Fig. 5C, E–H, J, K, M–O); pattern III, expressed in string-shaped tissues penetrating from the cortex to the inner medulla, including *m/z* 896.61 (t18:0-C22:0h) and 924.64 (t18:0-C24:0h; Fig. 5L, R). The string-shaped tissues were suggested to be collecting ducts; and pattern IV, expressed only in the inner medulla, including *m/z* 918.67 (d20:1-C24:0; Fig. 5P).

**Table 3:** Localization of the main sulfatide molecular species

*m/z* of Precursor [M − H]^-^	Predicted ceramide structure	Cortex	Outer medulla	Inner medulla	Collecting duct only	Category
778.51	d18:1-C16:0	〇	〇	〇		I
850.57	d18:1-C20:0h	〇	〇			II
862.60	d18:1-C22:0	〇	〇	〇		I
864.58	d18:1-C21:0h	〇	〇			II
876.58	d18:1-C22:1h	〇	〇			II
878.60	d18:1-C22:0h	〇	〇			II
880.61	d18:0-C22:0h	〇	〇			II
890.63	d18:1-C24:0/d20:1-C22:0	〇	〇	〇		I
892.61	d18:1-C23:0h	〇	〇			II
894.63	d18:0-C23:0h	〇	〇			II
896.61	t18:0-C22:0h				〇	III
904.61	d18:1-C24:1h	〇	〇			II
906.63	d18:1-C24:0h	〇	〇			II
908.65	d18:0-C24:0h	〇	〇			II
918.67	d20:1-C24:0			〇		IV
920.63	d20:0-C24:0	〇	〇	〇		I
924.64	t18:0-C24:0h				〇	III

The most abundant sulfatide species, including *m/z* 878.60, 892.61, and 906.63, containing ceramide composed of sphingosine (d18:1) and hFAs, were highly expressed in the outer medulla and some tubules in the cortex (Fig. 5G, J, N), whereas sulfatide species containing hFAs were not expressed in the inner medulla (Fig. 5C, E–H, J, K, M–O). Sulfatide species containing phytosphingosine (t18:0) were present over the entire length of the collecting ducts (Fig. 5L, R). Sulfatide species with C20-sphingosine were clearly detected in the inner medulla collecting ducts (IMCDs) (Fig. 5I, P) in accordance with a previous report (30).

### Sulfatide species of *m/z* 896.61 and 924.64 were present in particular cells along the collecting duct

**Fig. 6:**
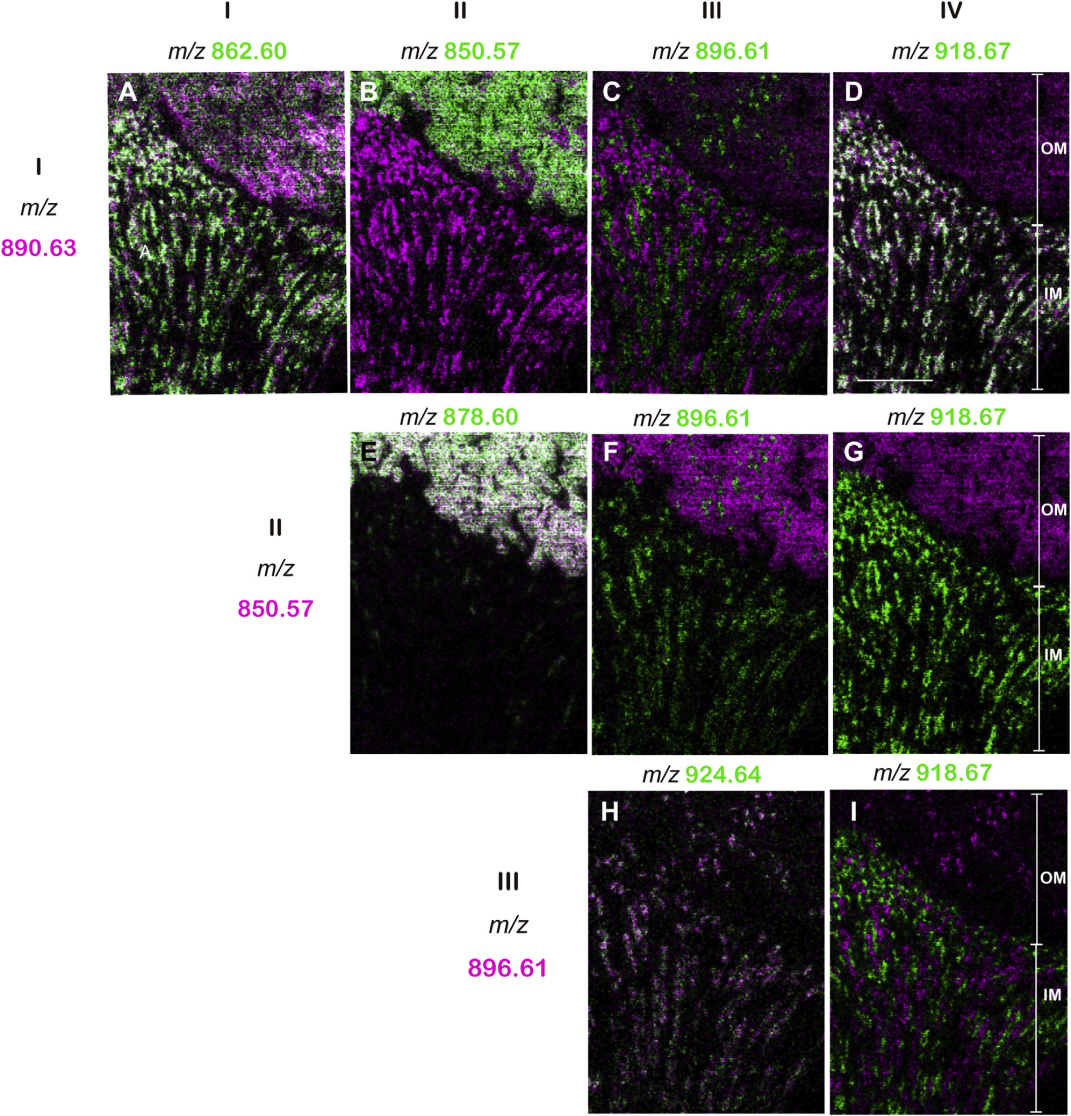
Comparison of the localization of sulfatide molecular species by IMS. A: Merged IMS images of patterns I (*m/z* 862.60, 890.63), II (*m/z* 850.57, 978.6), III (*m/z* 896.61, 924.64), and IV (*m/z* 918.67). Sulfatide molecules that showed pattern I distribution (*m/z* 890.63, magenta) were colocalized with pattern I (*m/z* 862.60, green in A) in the OM and IM, pattern II (*m/z* 850.57, green in B) in the OM, and pattern IV (*m/z* 918.67, green in D), but not pattern III (*m/z* 896.61, green in C). Molecules that showed a pattern II distribution (*m/z* 850.5, magenta in E and *m/z* 878.60, green in E) colocalized with each other. Molecules showing a pattern II distribution did not colocalize with pattern III (*m/z* 896.61, green in F) or pattern IV (*m/z* 918.67, green in G). Molecules that exhibited a pattern III distribution (*m/z* 896.61, magenta in H) and pattern III distribution (*m/z* 924.64 green in H) were colocalized in the IM and OM. Molecules that showed a pattern III distribution (*m/z* 896.61, magenta in I) and pattern IV distribution (*m/z* 918.67, green in I) were localized in different regions in the IM. The scale bar represents 200 μm. C, cortex; IM, inner medulla; OM, outer medulla.

To investigate which molecular species were colocalized, IMS images of specific sulfatide species were superimposed on each other. When sulfatide species with *m/z* 890.63 (d18:1-C24:0 and d20:1-C22:0; Fig. 5I) and *m/z* 862.60 (d18:1-C22:0; Fig. 5D), which were both expressed in the entire medulla and showed pattern I distribution, were colocalized in the inner and outer layers of the medulla (Fig. 6A). Furthermore, species with *m/z* 890.63 also colocalized with *m/z* 850.57 (d18:1-C20:0h; Figs. 5C and 6B), which showed pattern II distribution in the outer medulla, and with *m/z* 918.67 (d20:1-C24:0; Figs. 5P and 6D), which showed pattern IV distribution (Fig. 5, Fig. 6P) in the inner medulla. Sulfatide species of *m/z* 850.57 (Fig. 5C) and 878.60 (d18:1-C22:0h; Fig. 5G), which were both expressed in the outer medulla and showed pattern II distribution, were colocalized (Fig. 6E). However, the sulfatide species of *m/z* 850.57 was not colocalized with that of *m/z* 896.61 (d18:1-C22:0h; Figs. 5L and 6F) or *m/z* 918.67 (Fig. 6G). When sulfatide species with *m/z* 896.61 (t18:0-C22:0h; Fig. 5L) and *m/z* 924.64 (t18:0-C24:0h; Fig. 5, Fig. 6R), which both showed a pattern III distribution, were merged, colocalization was detected (Fig. 6H). As shown in Fig. 6I, the localization of sulfatide species of *m/z* 896.61 (t18:1-C22:0h) was not consistent with that of *m/z* 918.67 (d20:1-C24:0). These results suggested that sulfatide species with *m/z* 896.61 (t18:1-C22:0h) and *m/z* 924.64 (t18:0-C24:0h) may be characteristically expressed in some special cells along the collecting duct.

### Sulfatide species of *m/z* 896.61 and 924.64 were expressed in intercalated cells

**Fig. 7:**
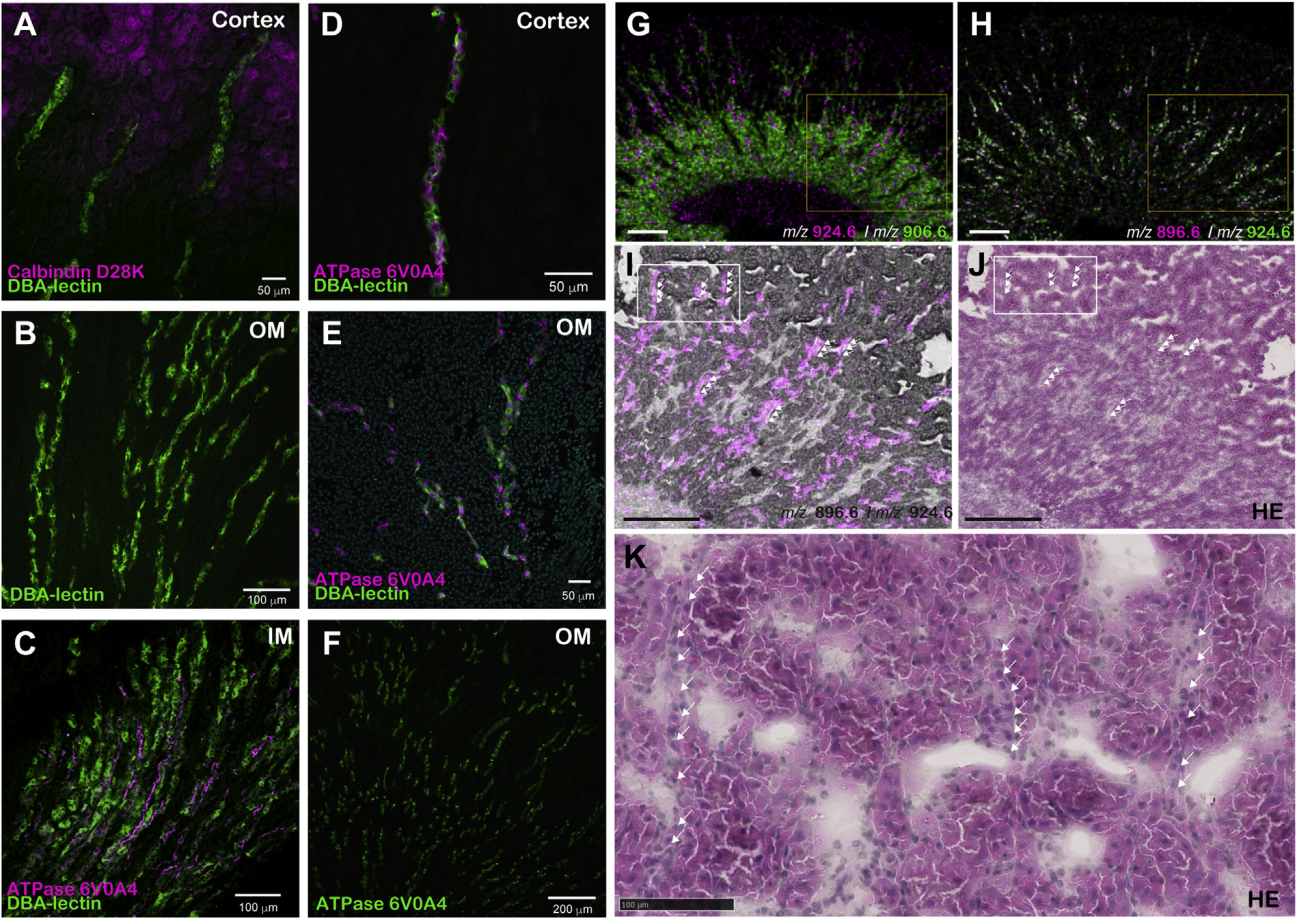
Comparison of the localization of sulfatide molecular species by immunohistochemistry and hematoxylin-eosin staining in mice. Immunohistochemical imaging of the collecting ducts in the cortex, OM, and IM (A-F). Distal tubule marker: calbindin D28K (magenta in A), main cells of the collecting duct: DBA-lectin (green in A–E) and intercalated cells of the collecting duct: ATP6VOA4 (magenta in C–E, F). Imaging of the collecting ducts in the cortex (A, D), OM (B, E, F), and IM (C). Merged IMS image of patterns II (*m/z* 906.6; green in G) and III (*m/z* 924.6; magenta in G) or patterns II (*m/z* 896.6; magenta in H) and II (*m/z* 924.6; green in H) with each other (G, H). Magnified images of the yellow-boxed areas in (G, H) are shown in (I, J). The pattern II sulfatide ion image at *m/z* 906.6 (green in G) and 924.6 (magenta in G) and the optical image were overlaid (I). Hematoxylin-eosin staining after IMS analysis is shown (J). Magnified image of the white enclosures in regions (I, J) is shown in (K). The white arrows (I, J, K) indicate the ion localization in pattern II. G-K were same sections. The scale bars in G and H represent 600 μm. The scale bars in I and J represent 500 μm. The scale bar in K represents 100 μm. IM, inner medulla; OM, outer medulla.

There are two types of cells along the collecting duct, that is, principal cells and intercalated cells. The ratio of principal to intercalated cells varies between tubule segments, with a ratio of 2:1 in the outer medullary collecting duct and 3:1 in the cortical collecting duct (16, 17, 18, 19). To investigate which types of cells expressed the sulfatide species of *m/z* 896.61 and 924.64, we evaluated IMS images and biomarker expression (Fig. 7). The lectin DBA binds to the apical aspect of the collecting duct (31, 32). The collecting ducts stained with DBA were distinguished from the distal convoluted tubules, which express calbindin (Fig. 7A). String-shaped tissues were stained from the cortex through the outer medulla to the inner medulla with DBA (Fig. 7A–C). Principal cells stained with DBA and intercalated cells expressing V-ATPase 6V0A4 (16, 17) were exclusive to each other along the same collecting duct (Fig. 7C, D). V-ATPase-positive intercalated cells were scattered around the collecting duct (Fig. 7C–F), similar to images of sulfatide species with *m/z* 896.61 and 924.64 (Fig. 6C, F, H, 7G, H).

On the other hand, when IMS images were compared, *m/z* 906.63 of pattern II and *m/z* 924.64 of pattern III were exclusive to each other from the cortex to the outer medulla and the population of *m/z* 924.64 ion signal positive cells were less than *m/z* 906.63 (Fig. 7G), indicating a similar localization to that of immunostaining. When histological images stained with hematoxylin-eosin overlapped with the IMS image, sulfatide species with *m/z* 896.61 and 924.64 of pattern III were found to be localized along a series of columnar epithelium penetrating from the cortex to the inner medulla, representing the collecting duct (Fig. 7I–K). Collectively, these findings of the similarities between V-ATPase 6V0A4 immunostaining (Fig. 7C–F) and the IMS images of *m/z* 896.61 and 924.64 (Fig. 6C, F, H, 7G, H) suggested that sulfatide species of *m/z* 896.61 and 924.64 were expressed in intercalated cells.

### Sulfatide species of *m/z* 896.61 and 924.64 were the predominant species in intercalated cells

**Fig. 8:**
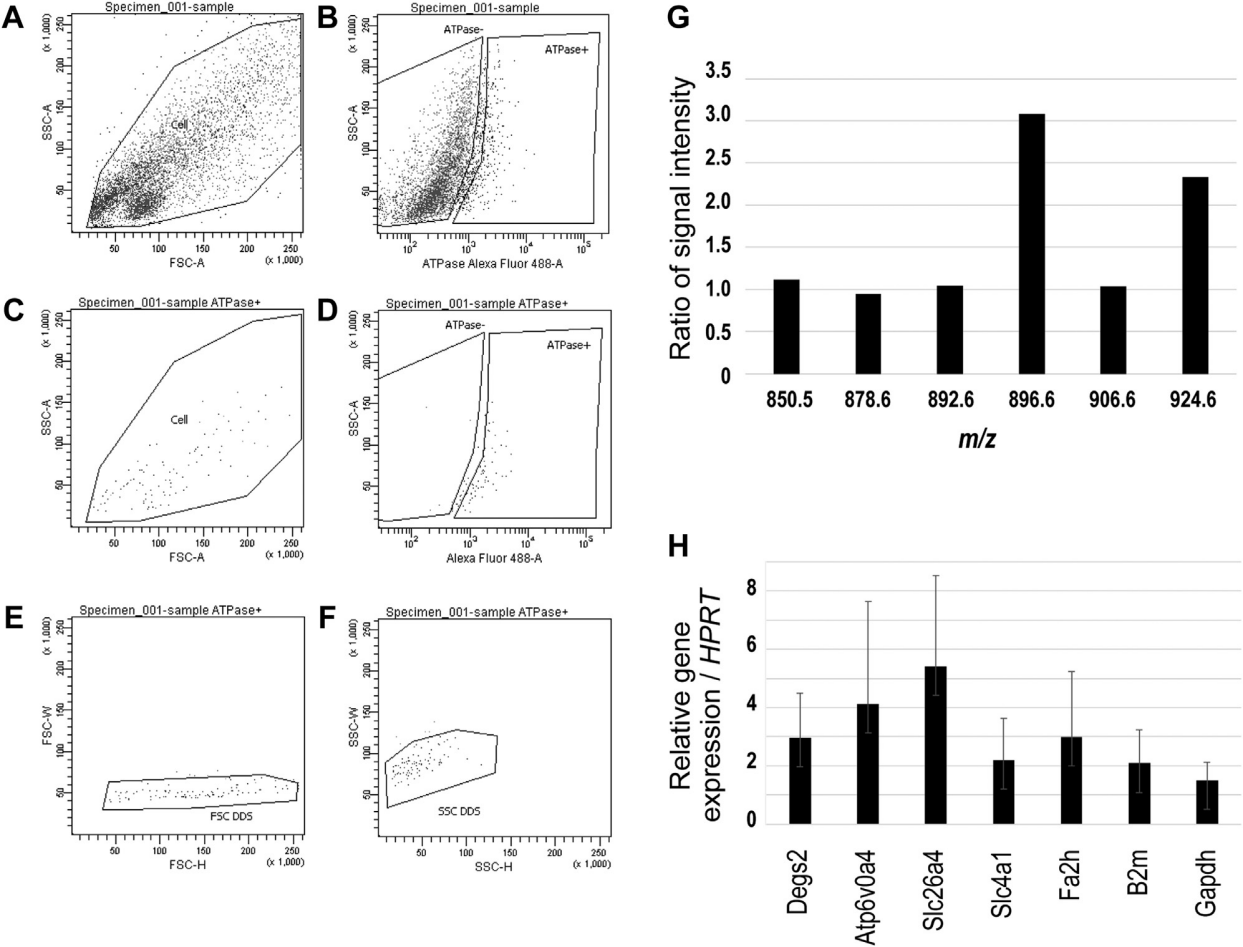
Expression of sulfatide species with phytosphingosine (t18:0) and 2-hFAs and the genes of corresponding enzymes in intercalated cells. Cells collected from collagenase-treated kidney tissues were labeled with anti-ATP6V0A4 antibodies, and the ATP6V0A4 high population was collected using a FACS Aria flow cytometer (A-F). Fractionated ATP6V0A4-positive doublet cells (B, D) were removed using FSC-W, FSC-H (E) and SSC-W, SSC-H (F). The sulfatide species in methanol extracts of the sorted ATP6V0A4-high population were analyzed using iMScope (G). Relative ratios of ion intensity for each sulfatide species in the ATP6V0A4-high population to that of the ATP6V0A4-low population, normalized to that of the PI ion (*m/z* 885.50), are shown (G). The ratio of *m/z* 896.61 and 924.64 ions in this ATP6V0A4-high population were increased in three independent experiments. The expression levels of *Dsgs2* (encoding sphingolipid delta(4)-desaturase) and *Fa2h* (encoding FA 2-hydroxylase) in the ATP6V0A4-high population were analyzed using RT-qPCR (H). *Atp6v0a4* (encoding ATP6V0A4 in intercalated cells), *Slc26a4* (encoding pendrin in type B-intercalated cells), and *AE1* (encoding SLC4A1 in type A intercalated cells) were used as positive controls. *B2m* (encoding b2-microglobulin) and *Gapdh* (encoding glyceraldehyde-3-phosphate dehydrogenase) were used as housekeeping genes.

To confirm whether sulfatide species of *m/z* 896.61 and 924.64 were expressed in intercalated cells, purified intercalated cells obtained using FACS were analyzed with MS. Collagenase-digested cells from murine kidneys were stained with primary antibodies recognizing V-ATPase 6V0A4, a marker for intercalated cells, stained with a fluorescently tagged secondary antibody, and then subjected to FACS analysis (Fig. 8A–F). The cells were sorted using the forward scatter and side scatter (Fig. 8A). And then, the sorted cells were divided into two populations—ATPase− (ATPase low) and ATPase+ (ATPase high) (Fig. 8B). The ATPase+ population were purified by eliminating ATP-positive doublet cells (Fig. 8C-F). Methanol extracts of purified V-ATPase 6V0A4-positive cells (Fig. 8D) were analyzed for sulfatide species using iMScope (Fig. 8G). The predicted sulfatide ions were detected at *m/z* 850.57, 878.60, 892.61, 896.61, 906.63, and 924.64. The relative ratio of ion intensity for each sulfatide species showed that the V-ATPase 6V0A4-positive intercalated cells predominantly contained sulfatide species with *m/z* 896.61 and 924.64 (Fig. 8G). This result was consistent with findings of IMS image analysis (Fig. 7).

Sulfatide species of *m/z* 896.61 and 924.64 are composed of a unique ceramide structure (t18:0-C22:0h and t18:0-C24:0h, respectively) containing three hydroxy groups. Phytosphingosine biosynthesis occurs via the sphingolipid C4-monooxygenase activity of DEGS2 (Delta 4-Desaturase, Sphingolipid 2; EC: 1.14.18.5) (33). By contrast, FA 2-hydroxylation is catalyzed by FA2H (FA 2-hydroxylase; EC: 1.14.18.6) (34). Purified V-ATPase 6V0A4-positive cells obtained by FACS expressed *Atp6v0a4*, *Slc26a4*, and *AE1* mRNA, which are markers of intercalated cells (Fig. 8H). These results indicated that the ATP-positive fraction was intercalated cell. Because *Degs2* and *Fa2h* mRNA were expressed in the V-ATPase 6V0A4-positive cells (Fig. 8H), the intercalated cells may produce sulfatide species containing a unique ceramide moiety composed of phytosphingosine and hFAs.

### Sulfatide species containing ceramide of t18:0-C22:0h and t18:0-C24:0h were present in human kidneys

**Fig. 9:**
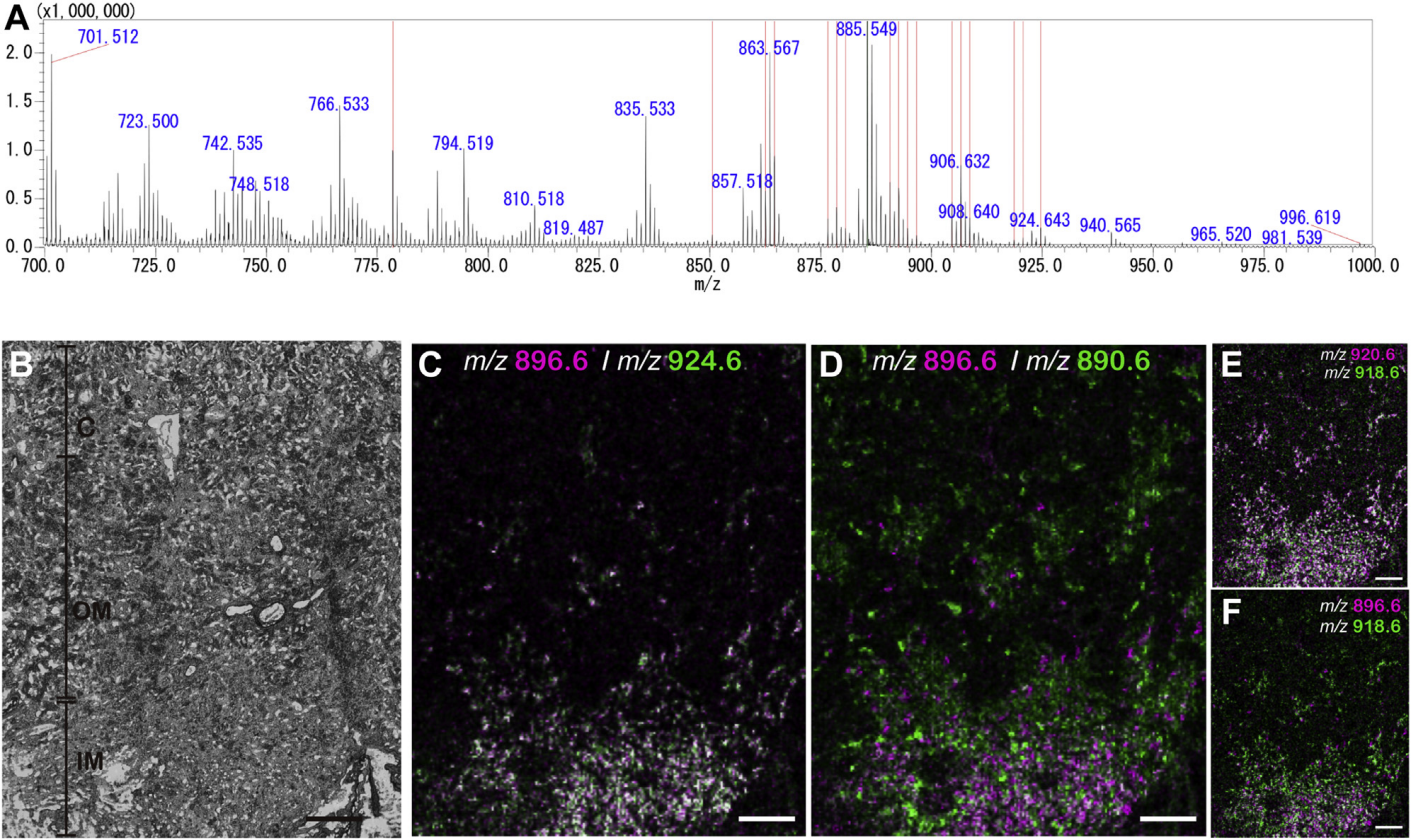
Mass spectra of the major sulfatide molecular species in the kidneys of humans and localization of the molecular species of *m/z* 896.6 and 924.6. MALDI-MS spectra of normal kidney tissues analyzed in negative ion mode with 9AA. The averaged mass spectra are shown. The red line shows the same spectral peaks as the 17 major sulfates found in mouse kidneys (A). Optical image of normal human kidney tissue (B). Sulfatide molecules of *m/z* 896.6 (pattern III, magenta) and 924.6 (pattern III, green) were colocalized with each other in the IM and OM (C). Molecules of *m/z* 896.6 (pattern III, magenta) and 890.6 (pattern I, green) showed different localizations in the IM and OM (D). Imaging of *m/z* 920.6 (pattern I, magenta) and 918.6 (pattern IV, green) showed colocalization in the IM and OM (E). Molecules of *m/z* 896.6 (pattern III magenta) and 918.6 (pattern IV, green) showed different localizations in the IM and OM (F). The scale bar represents 700 μm. C, cortex; IM, inner medull; OM, outer medulla.

Next, we investigated whether sulfatide molecular species of *m/z* 896.6 (t18:0-C22:0h) and 924.6 (t18:0-C24:0h) specifically expressed in intercalated cells were common in human. All 17 sulfatide molecular species found in the mouse kidney were also observed in the human kidney (Fig. 9A). In addition, IMS images of specific sulfatide species in the human kidney (Fig. 9C–F) were similar to those in the mouse kidney (Fig. 5, Fig. 6, Fig. 7). Sulfatide species with *m/z* 896.6 and 924.6 were colocalized, showing punctate structures from the cortex through the outer medulla to the inner medulla (Fig. 9C). However, the distribution patterns of sulfatide species of *m/z* 890.6, 918.6, and 920.6 were different from those of *m/z* 896.6 and 924.6 (Fig. 9D–F). These results suggested that sulfatide species containing ceramide composed of t18:0-C22:0h and t18:0-C24:0h were specifically localized in intercalated cells in the human kidney.

### Precursors of SM4s t18:0-C22:0h and SM4s t18:0-C24:0h did not accumulate in the collecting duct

**Fig. 10:**
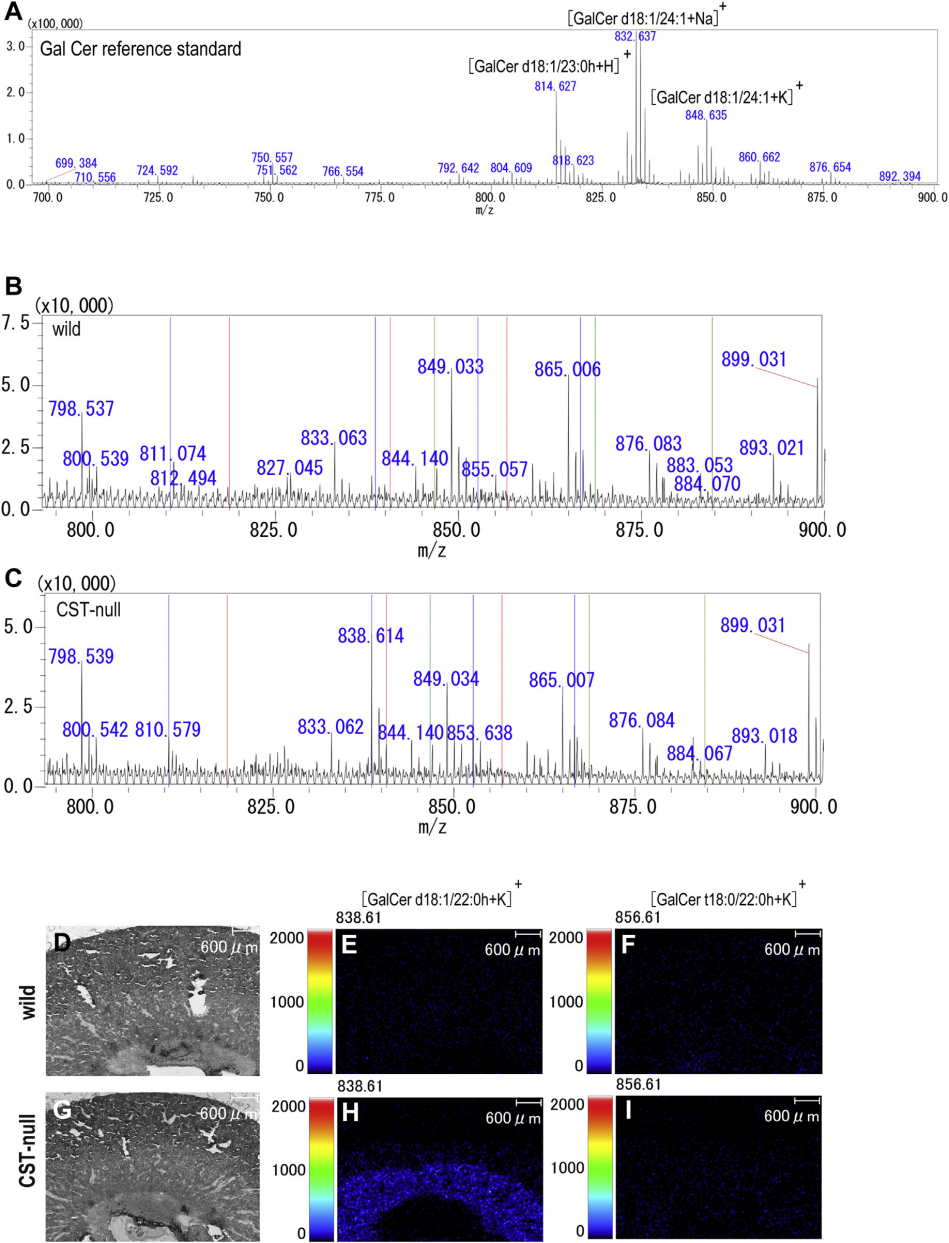
Mass spectra of galactosyl ceramide as a precursor of sulfatide by IMS. Reference standard of galactosyl ceramide derived from bovine analyzed in positive ion mode with CHCA (A). Spectrum in positive ion mode derived from wild-type (B) and *Cst*-null mouse kidneys (C). Red bars show the position of the precursors of SM4s t18:0-C22:0h at *m/z* 896.61, which are [GalCer t18:0-C22:0h+H]^+^ at *m/z* 818.6, [GalCer t18:0-C22:0h + Na]^+^ at *m/z* 840.63, and [GalCer t18:0-C22:0h + K]^+^ at *m/z* 856.61. Green bars show the position of the precursors of SM4s t18:0-C24:0h at *m/z* 924.64, which are [GalCer SM4s t18:0-C24:0h+H]^+^ at *m/z* 846.70, [GalCer SM4s t18:0-C24:0h + Na]^+^ at *m/z* 868.68, and [GalCer SM4s t18:0-C24:0h + K]^+^ at *m/z* 884.65. Blue bars show the [GalCer d18:1/20:0h + K]^+^ at *m/z* 810.58, [GalCer d18:1/22:0h + K]^+^ at *m/z* 838.61, [GalCer d18:1/23:0h + K]^+^ at *m/z* 852.63, and [GalCer d18:1/24:0h + K]^+^ at *m/z* 866.64, which were observed in the *Cst*-null mouse kidney. Representative images of GalCer ion obtained using IMS are shown (D-I). Optical image of wild-type (D) and *Cst*-null mouse (G). Ion localization of [GalCer d18:1/22:0h + K]^+^ at *m/z* 838.61 and [GalCer t18:0/22:0h + K]^+^ at *m/z* 856.61 in the wild-type (E, F) and *Cst*-null mice (H, I).

To investigate whether abnormalities of the intercalated cells in *Cst*-deficient mice occurred because of accumulation of the precursor of specific sulfatide species, we measured GalCer species in *Cst*-null mice using IMS (Fig. 10). In the GalCer reference derived from the bovine brain, three GalCer molecular species, namely [GalCer d18:1/23:0h+H]^+^ at *m/z* 814.6, [GalCer d18:1/24:1 + Na]^+^ at *m/z* 832.6, and [GalCer d18:1/24:1 + K]^+^ at *m/z* 848.6, were detected in the positive mode (Fig. 10A). No GalCer molecular species (Table 4) were detected in the wild-type mouse kidney (Fig. 10B). In contrast, four GalCer molecular species, namely [GalCer d18:1/20:0h + K]^+^ at *m/z* 810.58, [GalCer d18:1/22:0h + K]^+^ at *m/z* 838.61, [GalCer d18:1/23:0h + K]^+^ at *m/z* 852.63, and [GalCer d18:1/24:0h + K]^+^ at *m/z* 866.64, were detected in the *Cst*-null mouse kidney (Fig. 10C). Imaging analysis showed that these GalCer species accumulated in the inner medulla in the *Cst*-null mouse kidney (Fig. 10G, H). Neither precursor GalCer species with ceramide t18:0-C22:0h or t18:0-C24:0h was detected in the *Cst*-null mouse kidney (Fig. 10C, G, I). These results suggest that abnormalities in *Cst*-null intercalated cells are not because of the accumulation of precursors of sulfatide species (Table 4).

**Table 4:** GalCer precursors inferred from the 17 major sulfatide molecular species

Identified galactosyl ceramide	*m/z* of Precursor [M + H]^+^	*m/z* of Precursor [M + Na]^+^	*m/z* of Precursor [M + K]^+^
d18:1-C16:0	700.57	722.55	738.52
d18:1-C20:0h	772.62	794.61	810.58
d18:1-C22:0	784.66	806.64	822.62
d18:1-C21:0h	786.64	808.62	824.60
d18:1-C22:1h	798.64	820.62	836.60
d18:1-C22:0h	800.66	822.64	838.61
d18:0-C22:0h	802.67	824.65	840.63
d18:1-C24:0	812.69	834.67	850.65
d18:1-C23:0h	814.67	836.65	852.63
d18:0-C23:0h	816.69	838.67	854.64
t18:0-C22:0h	818.65	840.63	856.61
d18:1-C24:1h	826.67	848.65	864.63
d18:1-C24:0h	828.69	850.67	866.64
d18:0-C24:0h	830.70	852.68	868.66
d20:1-C24:0	840.72	862.71	878.68
d20:0-C24:0	842.70	864.68	880.66
t18:0-C24:0h	846.70	868.68	884.65

## Discussion

In this study, we demonstrated that there were morphological abnormalities in intercalated cells along the collecting duct in *Cst*-null kidneys and that sulfatide molecular species whose ceramide was composed of phytosphingosine (t18:0) and a very long-chain hFA were expressed in intercalated cells. Intercalated cells can be classified into three types: type A, type B, and non-A/non-B cells (15, 16, 17). Type A intercalated cells are present throughout the collecting duct and are morphologically characterized by numerous microprojections on a bulging apical surface, numerous mitochondria, a centrally located nucleus, and moderate basal infolding. V-ATPase is localized in the apical plasma membrane. Type B cells are mainly observed in the initial collecting duct and are characterized by intact nuclei and moderate basal infolding. The apical surface is less swollen than that in type A cells, and short microprojections are detected. V-ATPase is localized to the basolateral membrane. Finally, non-A/non-B cells are tall cuboidal cells and are mainly found in the connecting segment. These cells showed a surface covered with protruding microprojections, small vesicles, and mitochondria distributed in the cytoplasm. V-ATPase is localized in small vesicles and the apical membrane. However, in this study, we did not evaluate the specific type of intercalated cells expressing such unique sulfatide species. A study by Stettner *et al.* (13) suggested that NH_3_ excretion was suppressed through Rhcg in the apical aspect of type A intercalated cells, whereas other studies suggested that all types of cells along the collecting duct, including principal cells, collaborated functionally in the excretion of NH_3_ and protons to regulate acid-base homeostasis (15, 16, 17). Therefore, we could not exclude the possibility that other types of intercalated cells may also be involved in the suppression of NH_3_ excretion in the *Cst*-null kidney.

Recent studies have shown that under stress conditions such as metabolic acidosis, intercalated cells are adaptive and switch between type A and type B cells (35). In addition, transcriptional profiling analysis of single cells showed that the collecting duct generates various cell types via newly identified transitional cells (36). This transitional cell type expressed both intercalated cell and principal cell markers, suggesting that the cell types in the collecting duct undergo cell transitions that are altered by environmental influences (37, 38). Lithium treatment was reported to induce cellular remodeling of the collecting duct, ultimately increasing the ratio of renal intercalated cells to renal principal cells (39, 40). de Groot *et al.* (41) reported that a significant proportion of the proliferating cells of nuclear antigen-positive principal cells are arrested in the G2 phase of cell division. Himmel *et al.* (42) further suggested that the plasticity of renal IMCD cells may play an important role in lithium-induced renal IMCD remodeling. Therefore, further study using inducers of changes in the intercalated cell population such as chronic lithium exposure may directly demonstrate the consistent changes in the *m/z* 896.6 and 924.6 species of intercalated cells.

The existence of unique sulfatide species with ceramides consisting of a combination of 2-hFAs and phytosphingosine (t18:0) as the long-chain base has been reported in the kidneys using two-dimensional nuclear magnetic resonance (43) and MS (21). However, their distribution in the kidneys remains unknown. In this study, we evaluated the localization of these molecules in particular cells for the first time. As the scan pitch of IMS was 10–25 μm, it is difficult to confirm that these two molecules are completely expressed in the same cell. However, the ion signal in Fig. 6 clearly shows that the two molecules are in close proximity to each other in a large proportion of cases. Ceramides of these sulfatide species possess three hydroxy groups at the base of the lipid moiety corresponding to the interface between the plasma membrane and the extracellular environment under physiological circumstances. The hydroxylation of ceramides occurs in only a few types of glycosphingolipids, including GalCer and sulfatide. Because the GalCer synthase CGT is localized at the endoplasmic reticulum and prefers hFA-ceramides to non-hFA-ceramides, hFA-ceramides generated in the endoplasmic reticulum tend to be incorporated into GalCer (34). Subsequently, hFA-GalCer was sulfated to produce hFA-sulfatide by CST. Hydroxylation of glycosphingolipids facilitates lipid packing and influences the stability and permeability of the membrane by increasing the amount of hydrogen bonding at the interfacial region of the membrane (44). In fact, the hydroxylation of ceramides has been reported to affect membrane microdomains by strengthening the lateral interactions between neighboring proteins and lipids (45). Because strict control is required for the transport of small molecules, such as NH_3_ and protons, across the cell membrane, leak-proof structures of the plasma membrane are desirable.

Sulfation is essential for the NH_3_ transport activity of Rhcg, as demonstrated in a study of *Cst*-null kidneys (13); however, it is unknown whether hydroxylation of ceramide is necessary for it. The phytosphingosine structure is synthesized by the sphingolipid C4-monooxygenase activity of DEGS2 (33), and FA 2-hydroxylation is catalyzed by FA2H (34). *Fa2h*-deficient mice lack 2-hydroxylated sphingolipids in the central and peripheral nervous systems (46). Although normal compact myelin was formed in these mice, defects in 2-hydroxylated GalCer in the myelin membrane lead to loss of the long-term stability of myelin and eventual demyelination (46). 2-Hydroxylated sphingolipids are abundant, and FA2H is highly expressed in mammalian skin. In contrast to the nervous system, biosynthesis of most 2-hydroxylated sphingolipids was not influenced in *Fa2h*-null skin (47). In murine skin, FA2H is only expressed in sebaceous glands, and only a subset of 2-hydroxylated FAs (C20:0h, C22:0h, C23:0h, and C24:0h) incorporated into glucosylceramide is synthesized by FA2H (47). The set of 2-hydroxylated long-chain FAs synthesized by FA2H in the skin is utilized in sulfatide species in the kidneys. To the best of our knowledge, there are no reports on the physiological functions of the 4-hydroxylation of the sphingoid base. Although the *DEGS2* gene general function in the nervous system is unknown, a genome-wide association study of cognitive dysfunction in schizophrenia patients found an association with missense mutations in the *DEGS2* gene (48). The *DEGS2* gene is expressed in the brain, lungs, intestines, skin, and kidneys (49).

In intercalated cells of the kidneys, proton secretion is regulated by the V-ATPase complex, which is present in the lysosomal membrane and the apical aspect of the plasma membrane (50). In the current study, V-ATPase-positive vesicles were found to accumulate in intercalated cells. Therefore, the intracellular vesicles accumulated in intercalated cells were considered V-ATPase-containing lysosomes. NH_3_ accumulated in the cells may impair lysosomal function by increasing the intralysosomal pH, resulting in accumulation of lysosomes in the cells, similar to the pharmaceutical effects of chloroquine (51, 52). Alternatively, transportation of the V-ATPase protein to the apical membrane may be hampered owing to defects in sulfatide. When the trafficking and regulation of V-type ATPase are impaired by suppression of basolateral anion exchange activity, the size and number of type A intercalated cells are altered, and accumulation of lysosome-like vesicles and multilamellar vesicles is observed (53).

Several studies have demonstrated the involvement of specific sulfatide species in a variety of biological contexts. C18:0 and C18:0h sulfatide species are increased in the white matter of the neonatal rat brain after nitric oxide inhalation as a therapy for brain injury (54, 55). These C18:0 and C18:0h sulfatide species also appear in defined regions of oligodendrocyte generation, whereas sulfatide species with longer FAs, such as C24:0 and C24:0h, appear during oligodendrocyte maturation (25). The length of the FA chains of glycosphingolipids affects the fluidity of cell membranes (56) and possibly their biological functions. For example, the longer acyl chains of glycolipids are more readily recognized by antibodies because of increased exposure of the carbohydrate head group on the membrane surface (57). Aggregation based on sugar-sugar interactions between GalCer-containing liposomes and sulfatide-containing liposomes depends on hydroxylation and the length of the FA composition of sulfatide species (58). By contrast, a short-chain FA C16:0-containing sulfatide is predominant in the pancreas (59). C16:0 sulfatide inhibits glucose-induced insulin secretion by reducing the K^+^ channel sensitivity to the ATP block, whereas C24:0 sulfatide does not affect this process (58).

In conclusion, our findings showed that V-ATPase-positive vesicles accumulated in the cytoplasm of intercalated cells in the collecting duct of *Cst*-null kidneys. IMS analysis suggested that sulfatide molecular species with ceramide composed of t18:0-C22:0h and t18:0-C24:0h, which were characteristically expressed in intercalated cells, were involved in the excretion of NH_3_ and protons into the urine.

## Data availability

All data are contained within the article and available from the corresponding author upon reasonable request.

## Conflict of interest

The authors declare that they have no conflicts of interest with the contents of this article.
